# Decontamination of MDA Reagents for Single Cell Whole Genome Amplification

**DOI:** 10.1371/journal.pone.0026161

**Published:** 2011-10-20

**Authors:** Tanja Woyke, Alexander Sczyrba, Janey Lee, Christian Rinke, Damon Tighe, Scott Clingenpeel, Rex Malmstrom, Ramunas Stepanauskas, Jan-Fang Cheng

**Affiliations:** 1 Department of Energy Joint Genome Institute, Walnut Creek, California, United States of America; 2 Bigelow Laboratory for Ocean Sciences, Boothbay Harbor, Maine, United States of America; Université Paris-Sud, France

## Abstract

Single cell genomics is a powerful and increasingly popular tool for studying the genetic make-up of uncultured microbes. A key challenge for successful single cell sequencing and analysis is the removal of exogenous DNA from whole genome amplification reagents. We found that UV irradiation of the multiple displacement amplification (MDA) reagents, including the Phi29 polymerase and random hexamer primers, effectively eliminates the amplification of contaminating DNA. The methodology is quick, simple, and highly effective, thus significantly improving whole genome amplification from single cells.

## Introduction

The large amounts of DNA required for microbial genome sequencing are traditionally harvested from laboratory cultures, yet most microorganisms cannot be easily grown in isolation. Thus, the metabolic information encoded within most species is largely inaccessible with standard genomic approaches. Single cell whole genome amplification (WGA), however, circumvents this requirement for isolation by producing billions of genome copies from a single template. Multiple displacement amplification (MDA) using phi29 polymerase and random hexamer primers has become the preferred method for single cell WGA, and has successfully enabled partial and full genome recovery of microbes from a variety of environments [1]–[6]. However, the commercially available MDA reagents are frequently contaminated with unwanted DNA that is co-amplified with the target DNA, which reduces sequence efficiency and could confound analysis of unknown microbial genomes [6], [7]. While it is possible to prepare high purity Phi29 polymerase in house with careful measures of eliminating contaminating nucleic acids in many steps [7], a simpler and equally effective method of removing contaminants from commercial reagents has not been fully explored.

UV-irradiation can cause DNA single- and double-strand breaks, photooxidation damage of bases, and the formation of cyclobutane pyrimidine dimers [8]–[11]. These UV-induced lesions are inhibitory to DNA replication as the polymerase terminates or stalls at the lesion sites. Due to its simplicity, UV-irradiation has been used to treat PCR and MDA reagents to successfully suppress the amplification of unwanted DNA when dealing with single or a few copies of target DNA [3], [12], [13]. In the attempt of standardizing the UV-irradiation method, we here report the effect of different UV dosages on removing contaminant DNA from the MDA amplification reagents used for single cell whole genome amplification, as well as the UV impact on the enzymatic activity. From the analysis of genomic sequence data of >100 *Escherichia coli* single cells, we demonstrate the optimal range of UV treatment of MDA reagents for efficiently removing contaminant DNA without a significant reduction of the Phi29 activity or introducing additional single cell genome coverage bias or artifacts.

## Results and Discussion

**Figure 1:**
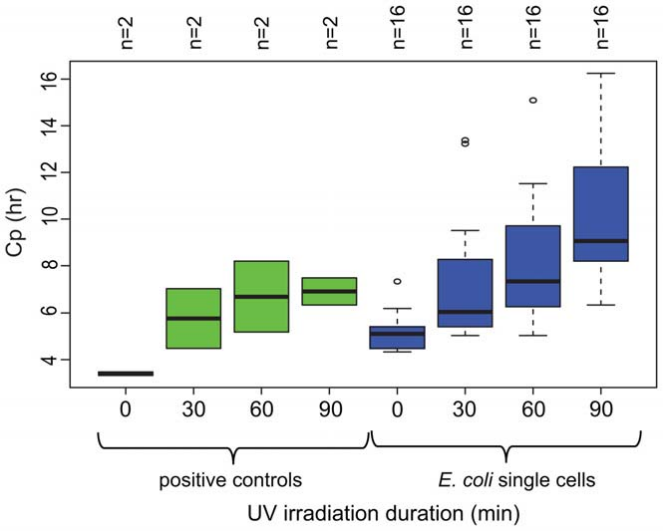
Crossing point (Cp) values for the real-time MDA of single *E. coli* cells and positive controls using unspiked MDA reagents UV-irradiated for 0, 30, 60 and 90 min.

Real-time MDA and high throughput shotgun sequencing allowed us to identify the ideal UV exposure required to eliminate exogenous DNA amplification while maintaining sufficient polymerase activity for whole genome amplification. Removal efficiency was assessed by intentionally contaminating MDA reagents with 50 fg of *Bacillus subtilis* DNA in each reaction, which is equivalent to approximately 10 genome copies. Contaminated and uncontaminated MDA reaction cocktails were irradiated for 0, 30, 60 and 90 min prior to real-time amplification of individual *E. coli* cells (Figures S1, S2, and S3). Amplification kinetics in the real-time MDA reactions of these single cells and positive controls (reactions with 10–100 *E. coli* cells) were compared between the UV-irradiations (Figure 1, Figure S3). We observed an increase of time required to amplify positive controls and single cells with an increase of UV treatment time. Only a marginal reduction of the number of amplified single cells and their fluorescent intensities of the final amplified products were observed if the UV treatment time was limited to 60 min. Most of the single cell amplified products represent approximately 10^8^-fold increase of DNA quantity (i.e. from 5 fg to 0.5 µg). In contrast, a much larger impact was seen with the amplification of background contaminated DNA in the real-time MDA curves. These amplification curves indicate a window of opportunity to harvest the amplified target genomes prior to the occurrence of background amplification. The observed deterioration of the MDA activity was due to the reduction of the Phi29 enzymatic activity as the MDA activity can be restored by adding more polymerase suggesting that the hexamers, nucleotides and other components are not the limiting factors in the UV treated reagents (data not shown). In summary, the real-time MDA data suggests that the 60 min UV treatment of the reagents effectively eliminates amplification in no template controls and does not have a significant impact on the polymerase activity in single cell reactions.

**Figure 2:**
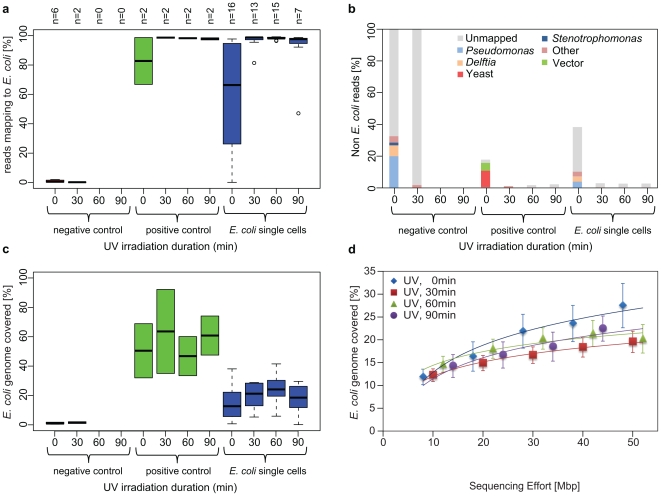
Shotgun sequence analysis of single *E. coli* cells amplified with MDA reagents that were UV irradiated for 0, 30, 60 and 90 min (A–C). Green boxplots represent positive controls and blue boxplots single *E. coli* cells (A, B). The box is drawn between the first and third quartiles, with the thick black lines representing the median. Dotted lines extend to the minimum and maximum values and outliers are shown as circles. In untreated samples, a large number of sequences mapping to *Pseudomonas*, *Delftia* and *Stenotrophomonas* genomes were found in no template controls (negative controls), as wells a substantial number unmappable reads, which may represent self priming of random hexamers. With 60 min UV irradiation, the contamination in the negative controls was successfully eliminated, leaving no DNA for library generation. Positive controls (10–100 *E. coli* cells) and individual *E. coli* cells were free of contamination after 30 min of UV treatment, with ∼98% (median) of reads mapping to the *E. coli* genome and covering approximately 64% and 21% respectively, which is to be expected at the given sequence effort. (D) Genome coverage rarefaction analysis for the 51 *E. coli* single cells (UV 0 min, n = 16, UV 30 min, n = 13, UV 60 min, n = 15, UV90 min, n = 7) sequenced shows no significant difference with treatment durations, suggesting that UV irradiation did not negatively impact on the genome recovery. Error bars represent std errors.

To verify our real-time MDA results, we performed shotgun sequencing of 109 *E. coli* single amplified genomes and 37 control samples on the Illumina GAIIX platform (Figure S1). We generated 7.6 Gbp from these libraries, which corresponds to approximately 10x sequence coverage for each MDA product (Supplementary Methods). Reads were mapped to the *E. coli* and *B. subtilis* genomes as well as blasted against the nt database to determine the composition of the sequencing libraries. We found that a 60 min UV treatment (or an accumulative dose of 11.4 J/cm^2^) of the MDA reagents completely eliminated the common contaminants (e.g. *Pseudomonas* and *Delftia* sequences) typically found in untreated samples (Figure 2A–C). Even with the 30 min UV treatments, most of the common contaminants were removed from the reagents. We also observed a bias of unmapped reads (i.e. no similarity to any GenBank organisms) surviving the UV treatments even as high as 90 min (an accumulative dose of 17 .1 J/cm2). Unmapped reads could either represent contaminated organisms that have not been sequenced yet or the elongated products of hexamers priming each other. The lack of both, sequence similarity (blastx hits) to known proteins and predicted long open reading frames (ORFs), as well as the absence of matching reads amongst different UV-treated samples (data not shown) suggest that these unmapped reads originated from random hexamers. Similarly, the percentage of reads matching *B. subtilis* in intentionally contaminated libraries dropped to nearly zero after 60 minutes of exposure (Figure S4).

To assess whether the UV treatment diminished genome recovery, we generated rarefaction curves of genome coverage for the single cell genome assemblies (Figure 2D). Rarefaction curves for the different treatment durations did not show significant difference, suggesting that UV treatment does not systematically impact genome recovery. Twelve single *E. coli* cells were sequenced to a greater depth (∼160x sequence coverage), yielding genome recovery of ∼32–72% based on read mapping or 13–41% when using de novo assembly. These estimates provide a baseline on what one can expect to recover from a single cell given the protocols used in this study and a short-read sequence depth of 160x (Figure S5). We moreover assessed the impact of the photo-damaged hexamers to the error rate of the amplified genomes. The average error rate of the resulting E. coli reads was not significantly different for the different UV treatments: 1.1±0.1%, 0.9±0.1%, 0.9±0.1%, and 0.8±0.2% for UV treatments of 0, 30, 60, and 90 minutes, respectively. This result indicates either the photodamaged hexamers were not incorporated into the amplified genomes or the UV treatment does not impact the enzyme's proofreading ability. Thus, UV irradiation is a simple and effective treatment for decontaminating MDA reagents used for single cell genome amplification.

## Materials and Methods

### Single-cell sorting

The cells used in this study, *Escherichia coli* str. K-12 substr. MG1655 (TaxID: 511145), were originally obtained from ATCC (strain #700926). Cells were collected following the clean sorting procedures detailed by Rodrigue et al. 2009 [14]. Briefly, a stationary phase culture of individual *E. coli* cells was sorted by the Cytopeia Influx Cell Sorter (BD Biosciences) into two 96 well plates containing 3 µl of UV treated TE. The cells were stained with SYBR Green I (Invitrogen) and illuminated by a 488 nm laser (Coherent Inc.). The sorting window was based on size determined by side scatter and green fluorescence (531/40 bp filter). For each plate, single cells were sorted into eight columns, 100 and 10 cells into one column, a droplet of sheath fluid into one column (noise sort), and no droplets at all into two columns (no sort), for a total of one plate (Figure S2).

### Single cell lysis and real-time multiple displacement amplification (MDA)

We compared two procedures for UV decontamination of reagents: (i) non-spiked MDA reagents and (ii) spiked MDA reagents. *E.coli* single cells and controls in one 96-well plate were lysed for 20 min at room temperature using alkaline solution from the Repli-G UltraFast Mini Kit (Qiagen) according to manufacturer's instructions. After neutralization, the samples were amplified using the Repliphi Phi29 reagents (Epicentre). Each 50 µl reaction contained Phi29 Reaction Buffer (1X final concentration), 50 µM random hexamers with the phosphorothioate bonds between the last two nucleotides at the 3′ end d (IDT), 0.4 mM dNTP, 5% DMSO (Sigma), 10 mM DTT (Sigma), 100 U Phi29 and 0.5 µM Syto 13 (Invitrogen). A mastermix of MDA reagents minus the Syto 13 (degrades when exposed to UV) sufficient for a 96-well plate was assembled and then aliquoted into four Eppendorf Safe-Lock 1.5 ml clear microcentrifuge tubes. The tubes of mastermix were UV treated on ice (Figure S1) in the Stratalinker 2400 UV Crosslinker (Stratagene) at 254 nm for 0, 30, 60 and 90 min. These represent the UV doses of 0, 5.7, 11.4 and 17.1 J/cm^2^, respectively, when measuring inside the eppendorf tubes at the 4 cm distance to the light bulb (Figure S2). Syto 13 was added to the mastermix after UV treatment and each tube of treated MDA mastermix was added to one quarter of the 96-well plate of lysed *E.coli* single cells including respective controls (Figure S2). The MDA reactions were run in real time on the Roche LightCycler 480 for 17 hours at 30°C. The same procedure was used for a second 96-well plate, but the MDA mastermix was purposefully contaminated with the addition of 50 fg of *Bacillus subtilis* DNA per MDA reaction prior to UV treatment.

### Indexed Illumina library construction and sequencing

Single cell amplified DNA was sheared in 100 µl using the Covaris E210 with the setting of 10% duty cycle, intensity 5, and 200 cycle per burst for 6 min per sample. The concentration and fragment size of each sheared sample was determined on the Caliper GX machine using the manufacture's recommended conditions. The fragment sizes were in the range of 250 to 400 bp, and the concentration ranged from 0 to 37.25 ng/µl. The sheared DNA was end-repaired, A-tailed, and ligated to the Illumina adaptors according to the Illumina standard PE protocol. The adaptor-ligated samples were then amplified by PCR for 10 cycles using a set of 96 indexed primers. The concentration of the resulted 96 Illumina indexed libraries was again determined using the Caliper GX machine. Two nM of DNA fragments (0.5 to 12 µl) of each library were pooled together and the main library bands around 300 bp were gel-purified and dissolved in 30 µl TE. The two library pools, one spiked with *B. subtilis* DNA and one without, had a concentration of 21.5 ng/µl (or 105.9 nM) and 25.4 ng/µl (or 120.1 nM), respectively. One lane of flowcell was generated from each library pool and sequenced in an Illumina GAIIx sequencer according to the manufacturer's protocols. Approximately 4.1 and 3.5 Gbp of sequence data were collected from the spiked and unspiked pooled libraries, respectively. Another aliquot of 2 nM from a selected set of twenty indexed libraries derived from the unspiked plate were pooled together to form 4 new library pools. Approximately 8 Gbp of additional sequence data was generated from these 4 library pools to increase the sequence depth of these SAGs.

### Data analysis

Sequences derived from each SAG were mapped to reference genomes of *Escherichia coli K-12* (U00096.2), *Delftia acidovorans SPH-1* (CP000884.1), and 22 Pseudomonas genomes (including *Pseudomonas syringae* (NC_004578.1, NC_004632.1, NC_004633.1, NC_005773.3, NC_007005.1, NC_007274.1, NC_007275.1), *Pseudomonas putida* (NC_002947.3, NC_009512.1, NC_010322.1, NC_010501.1), *Pseudomonas fluorescens* (NC_004129.6, NC_007492.2, NC_009444.1, NC_012660.1), *Pseudomonas aeruginosa* (NC_002516.2, NC_008463.1, NC_009656.1, NC_011770.1), *Pseudomonas stutzeri* (NC_009434.1), *Pseudomonas mendocina* (NC_009439.1), and *Pseudomonas entomophila* (NC_008027.1)) using the short read alignment program bwa (version 0.5.8c, default mapping parameters) [15] to determine the fraction of reads mapping to each of the three groups. Unmapped reads were further compared to NCBI's non-redundant nucleotide database using megablast 2.2.23. The best BLAST hits were used to determine the distribution of phyla matched by the reads from each SAG.

Based on the alignments to *Escherichia coli K-12* and *de-novo* assemblies of all SAGs, we calculated the fraction of the reference genome covered by at least one read, and the contigs resulting from assembly, respectively (Figures 2, S4, and S5). The MDA amplification introduces a tremendous bias in the sequencing coverage of the genome causing problems in the assembly process. Therefore, all raw Illumina sequence data was passed through a filtering program developed at JGI, which filters out known Illumina sequencing and library preparation artifacts. Specifically, all reads containing sequencing adapters, low complexity reads, and reads containing short tandem repeats were removed. Duplicated read pairs derived from PCR amplification during library preparation were identified and consolidated into a single consensus read pair. The artifact filtered sequence data was screened and trimmed according to the k-mers present in the dataset. High-depth k-mers presumably derived from MDA amplification bias cause problems in the assembly, especially if the k-mer depth varies in orders of magnitude for different regions of the genome. We removed reads representing high-abundance k-mers (>32x k-mer depth, k = 31) and trimmed reads that contain unique k-mers.

The filtered reads of each SAG were assembled into contigs using Velvet version 1.1.04 [16]. The VelvetOptimiser script (version 2.1.7) was used with default optimization functions (n50 for k-mer choice, total number of base pairs in large contigs for cov_cutoff optimization).

Rarefaction analysis was performed by sub-sampling the BAM alignment files generated by bwa (see above). For each sample size an appropriate number of pairs of reads were extracted randomly from the BAM file where both reads mapped to the *E. coli* reference sequence. The mapping information of the sub-samples was used to calculate the fraction of the *E. coli* reference covered by at least one read. Additionally, we assembled each subsample and mapped the contigs back to the reference (Figure S5).

We also analyzed the error rate of the Illumina reads to assess whether UV treatment has any impact on Phi29 proof reading activity. BAM alignment files were used to calculate the number of exact matching bases, mismatches, insertion, deletions, and number of clipped bases (bwa soft clipping). For each *E. coli* single cell, error rates were calculated for all reads mapping to the *E. coli* reference genome.

## Supporting Information

Figure S1 **Experimental design.** Unspiked and spiked (50 fg of *B. subtilis* DNA per reaction as intentional contamination) multiple displacement amplification (MDA) reagents were UV treated for 0, 30, 60 and 90 min, then used to amplify sorted single *E. coli* cells and controls. The 96-well plate layout for single cell sorting and amplification included six negative controls (no template), two positive controls (10–100 cells) and 16 single cells per treatment (see Methods for more details). Wells that did not generate an MDA DNA product are marked in grey. Indexed Illumina libraries were constructed from each MDA product, followed by low-level shotgun sequencing at ∼10x coverage.
(DOCX)

Figure S2 **Schematic cross section of the UV irradiation setup.** We used UV treatment to eliminate possible contamination in MDA reagents prior to single cell whole genome amplification. Since high temperatures can inactivate the Phi29 polymerase, the tubes of MDA mastermix were UV irradiated on ice. The tubes were floated in 4C chilled MilliQ water in a reflective container (here pipette tip box lid lined with aluminum foil) and stationed at a distance of 8.5 cm from the UV bulb. The reflective container holding the water and mastermix was kept cool, surrounded by ice packs within an ice bucket. The entire apparatus was placed within the Stratalinker 2400 for the duration of the UV treatment.
(DOCX)

Figure S3 **Real-time MDA of single** ***E. coli*** **cells using (a) unspiked MDA reagents and (b) spiked MDA reagents, UV irradiated for 0, 30, 60 and 90 min.** Fluorescence was measured real-time for 17 hours to quantify the amount of DNA produced during MDA. Without any UV treatment of the MDA mastermix, no template controls generated substantial amounts of MDA product. With increasing UV irradiation times, DNA amplification in negative controls was suppressed, suggesting that contaminating DNA was successfully removed with a minimal effect on the overall amplification kinetics.
(DOCX)

Figure S4 **Shotgun sequence analysis for single** ***E. coli*** **cells amplified with** ***Bacillus subtilis*** **DNA spiked into MDA reagents prior to UV irradiation for 0, 30, 60 and 90 min.** Red boxplots represent negative controls, green boxplots positive controls and blue boxplots *E. coli* single cells. The box is drawn between the first and third quartiles, with the thick black lines representing the median. Dotted lines extend to the minimum and maximum values and outliers are shown as circles. With 60 min UV treatment, the contaminant (*B. subtilis* DNA) has largely been eliminated as suggested by the majority of the reads (median = 98.9%) mapping to the *E. coli* genome, while the median percentage of reads mapping to the *Bacillus* genome drop from 82.2% (no UV irradiation) to 0.5% (30 min UV irradiation) to 0.2% (60 min UV irradiation).
(DOCX)

Figure S5 **Genome coverage rarefaction analysis for 12** ***E. coli*** **single cells sequenced at ∼160x depth show the recovery of ∼32**–**72% of the genome at >/ = 1x coverage as based on read mapping (raw reads) and 13**–**41% when using de novo assembly (contigs).** Error bars represent std errors.
(DOCX)
