# Supplementary material for: Decontamination of MDA Reagents for Single Cell Whole Genome Amplification
Source: PLoS One. 2011 Oct 20;6(10):e26161. doi: 10.1371/journal.pone.0026161 (PMC3197606; doi:10.1371/journal.pone.0026161)
Supplement: Figure S1 — Experimental design. Unspiked and spiked (50 fg of B. subtilis DNA per reaction as intentional contamination) multiple displacement amplification (MDA) reagents were UV treated for 0, 30, 60 and 90 min, then used to amplify sorted single E. coli cells and controls. The 96-well plate layout for single cell sorting and amplification included six negative controls (no template), two positive controls (10–100 cells) and 16 single cells per treatment (see Methods for more details). Wells that did not generate an MDA DNA product are marked in grey. Indexed Illumina libraries were constructed from each MDA product, followed by low-level shotgun sequencing at ∼10x coverage. (DOCX) [file pone.0026161.s001.docx]

**Supplementary Figure S1**
