# Supplementary figures and images for: Decontamination of MDA Reagents for Single Cell Whole Genome Amplification
Source: PLoS One. 2011 Oct 20;6(10):e26161. doi: 10.1371/journal.pone.0026161 (PMC3197606; doi:10.1371/journal.pone.0026161)

**Supplementary Figure S2**


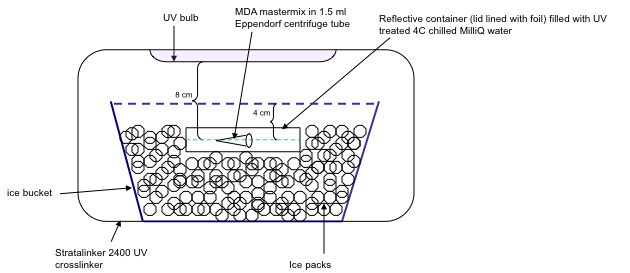

Supplement: Figure S2 — Schematic cross section of the UV irradiation setup. We used UV treatment to eliminate possible contamination in MDA reagents prior to single cell whole genome amplification. Since high temperatures can inactivate the Phi29 polymerase, the tubes of MDA mastermix were UV irradiated on ice. The tubes were floated in 4C chilled MilliQ water in a reflective container (here pipette tip box lid lined with aluminum foil) and stationed at a distance of 8.5 cm from the UV bulb. The reflective container holding the water and mastermix was kept cool, surrounded by ice packs within an ice bucket. The entire apparatus was placed within the Stratalinker 2400 for the duration of the UV treatment. (DOCX) [file pone.0026161.s002.docx]

**Supplementary Figure S5**


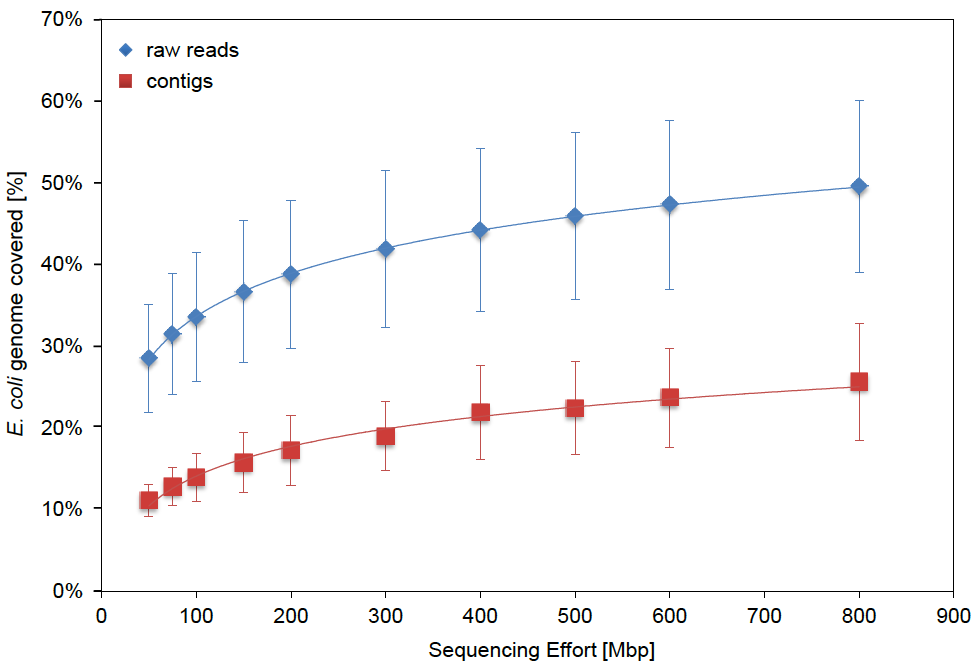

Supplement: Figure S5 — Genome coverage rarefaction analysis for 12 E. coli single cells sequenced at ∼160x depth show the recovery of ∼32–72% of the genome at >/ = 1x coverage as based on read mapping (raw reads) and 13–41% when using de novo assembly (contigs). Error bars represent std errors. (DOCX) [file pone.0026161.s005.docx]
