# Supplementary material for: Decontamination of MDA Reagents for Single Cell Whole Genome Amplification
Source: PLoS One. 2011 Oct 20;6(10):e26161. doi: 10.1371/journal.pone.0026161 (PMC3197606; doi:10.1371/journal.pone.0026161)
Supplement: Figure S4 — Shotgun sequence analysis for single E. coli cells amplified with Bacillus subtilis DNA spiked into MDA reagents prior to UV irradiation for 0, 30, 60 and 90 min. Red boxplots represent negative controls, green boxplots positive controls and blue boxplots E. coli single cells. The box is drawn between the first and third quartiles, with the thick black lines representing the median. Dotted lines extend to the minimum and maximum values and outliers are shown as circles. With 60 min UV treatment, the contaminant (B. subtilis DNA) has largely been eliminated as suggested by the majority of the reads (median = 98.9%) mapping to the E. coli genome, while the median percentage of reads mapping to the Bacillus genome drop from 82.2% (no UV irradiation) to 0.5% (30 min UV irradiation) to 0.2% (60 min UV irradiation). (DOCX) [file pone.0026161.s004.docx]

**Supplementary Figure S4**
